# Genome-Wide Identification, Expression Patterns, and Functional Analysis of UDP Glycosyltransferase Family in Peach (*Prunus persica* L. Batsch)

**DOI:** 10.3389/fpls.2017.00389

**Published:** 2017-03-22

**Authors:** Boping Wu, Liuxiao Gao, Jie Gao, Yaying Xu, Hongru Liu, Xiangmei Cao, Bo Zhang, Kunsong Chen

**Affiliations:** Zhejiang Provincial Key Laboratory of Horticultural Plant Integrative Biology/Laboratory of Fruit Quality Biology, Zhejiang UniversityHangzhou, China

**Keywords:** expression pattern, peach, phylogeny, secondary metabolites, UGT

## Abstract

Peach (*Prunus persica* L. Batsch) is a commercial grown fruit trees, important because of its essential nutrients and flavor promoting secondary metabolites. The glycosylation processes mediated by UDP-glycosyltransferases (UGTs) play an important role in regulating secondary metabolites availability. Identification and characterization of peach *UGTs* is therefore a research priority. A total of 168 peach *UGT* genes that distributed unevenly across chromosomes were identified based on their conserved PSPG motifs. Phylogenetic analysis of these genes with plant UGTs clustered them into 16 groups (A–P). Comparison of the patterns of intron–extron and their positions within genes revealed one highly conserved intron insertion event in peach *UGTs*. Tissue specificity, temporal expression patterns in peach fruit during development and ripening, and in response to abiotic stress UV-B irradiation was investigated using RNA-seq strategy. The relationship between *UGTs* transcript levels and concentrations of glycosylated volatiles was examined to select candidates for functional analysis. Heterologous expressing these candidate genes in *Escherichia coli* identified UGTs that were involved in the *in vitro* volatile glycosylation. Our results provide an important source for the identification of functional *UGT* genes to potential manipulate secondary biosynthesis in peach.

## Introduction

Glycosyltransferases (GTs; EC 2.4.x.y) are found in all living organisms, and are responsible for metabolic processes. GTs catalyze the transfer of sugar moieties from activated donor molecules to specific acceptor molecules such as sugars, lipids, proteins, nucleic acids, antibiotics, and small molecules. Information of GTs can be found in the Carbohydrate-Active enZymes database (CAZy^1^). Among the 101 GT families present in the CAZy database in December 2016, GT1 family is the largest one. GT1s are also referred to as UDP-glycosyltransferases (UGTs) because UDP-glucose can act as sugar donor. The plant UGTs are characterized by the presence of a highly conserved 44-amino acid C-terminal consensus sequence, termed the plant secondary product glycosyltransferase (PSPG) box (Vogt and Jones, 2000).

UDP-glycosyltransferases have been previously identified in various plant species, from the lowest plant such as *Chlamydomonas reinhardtii* to higher plants such as *Vitis vinifera* (Yonekura-Sakakibara and Hanada, 2011). Over 100 UGTs have been identified from *Arabidopsis thaliana* (Li et al., 2001), which could be clustered into 14 groups based on the amino acid sequences. Such high abundance of UGTs in plants demonstrates their indispensable roles in metabolism of natural products such as secondary metabolites. Arabidopsis *UGT79B1* and *UGT91A1* mediated anthocyanin modification (Yonekura-Sakakibara et al., 2012), similar function of *UGT* genes were also observed in strawberry (Song et al., 2016b), peach (Cheng et al., 2014), and kiwifruit (Montefiori et al., 2011). Diversity of bioactive flavonol glycosides was also due to UGTs catalyzed modifications (Ono et al., 2010). Apart from anthocyanin and flavonol, *UGTs* were involved in modification of flavor-related volatiles in plants, particular for fruit. Involvement of *UGTs* in biosynthesis of glycosylated volatiles were identified in developing fruit such as grapevine (Bönisch et al., 2014a,b), kiwifruit (Yauk et al., 2014), and strawberry (Song et al., 2016a). These odorless glycosylated volatiles can be liberated during fruit development and ripening through acid or enzymatic hydrolysis, releasing free volatiles and potentially influencing flavor quality. In tomato fruit, *NON-SMOKY GLYCOSYLTRANSFERASE1* (*NSGT1*) was the major determinant of flavor quality via glycosylation of phenylpropanoid volatiles, therefore preventing the release of smoky aroma (Tikunov et al., 2013).

Peach (*Prunus persica* L. Batsch) is a member of Rosaceae family, and is the third most important deciduous fruit trees worldwide. Recently, *UGT* genes were likely to be responsible for glycosylation of anthocyanins in peach flowers (Cheng et al., 2014). This observation prompts us to further explore and characterize the potential functions of peach *UGTs*. Here, we present a genome-wide analysis of *UGT* genes in peach based on availability of the genome database^2^. Location of *UGT* genes on chromosomes was analyzed, and their extron–intron architecture was compared. RNA-seq was carried out to investigate expression patterns of *UGT* genes in various organs of peach, during fruit development and ripening, and in response to abiotic stress. Moreover, peach *UGT* genes were heterologous expressed in *Escherichia coli* to study their potential functions such as formation of glycosylated volatiles.

## Materials and Methods

### Plant Materials and Treatment

Peach (*Prunus persica* L. Batsch cv. Hujingmilu) fruit, flowers, and leaves were obtained from the Melting Peach Research Institute of Fenghua, Zhejiang Province, China. Peach fruit were harvest at four stages (S1, S2, S3, and S4) according to previous study (Botton et al., 2016; Wang et al., 2016), representing the first fast growth (34 days after bloom, DAB, fruit weight = 5.69 ± 0.37 g), endocarp lignification (stone hardening, 71 DAB, 45.29 ± 0.63 g), the second fast growth (94 DAB, 113.93 ± 2.71 g), and mature stage (ready for harvest, 108 DAB, 207.48 ± 2.08 g), respectively. Peach fruit growth curve and photos of four stages were shown in **Supplementary Figure S1**. In our experiment, fruit with firmness = 26.70 ± 5.16 N, total soluble solids = 11.18 ± 0.68 ^o^Brix, lightness = 65.80 ± 0.44, hue angle = 100.99 ± 0.30 and chroma = 32.31 ± 0.22 were harvested at mature stage. After harvested at the S4 stage, fruit were allowed to ripen up to 6 days (20°C, 90–96% relative humidity), and were sampled at 3 days (S4 + 3d) and 6 days (S4 + 6d). At each sampling time, peach flesh tissues were collected. Vegetative tissue samples were taken from full expanded mature leaves, and flowers were collected at full blossom. For UV-B treatment, mature peach fruit were randomly divided into two groups, and were stored in climatic chambers without any natural light. One group fruit were exposed to irradiation of UV-B (280–315 nm) for 6 and 48 h at 20°C and relative humidity 90–96%. UV-B lamp tubes (Luzchem Research, Inc., Gloucester, ON, Canada) provided 1.50 w/m^2^ at fruit height (approximately 50 cm under the lamps). Control fruit were covered with aluminum foil to avoid exposure to light, and were placed next to the samples undergoing UV-B irradiation according to Suzuki et al. (2015). After treatment, slices of peel tissue (∼1 mm thick) were carefully separately. At each time point, three biological replicates with five fruit each were harvested. Peach tissues were immediately frozen in liquid nitrogen and then stored at -80°C until further analysis.

### Identification of Peach UGT Genes

A feature sequence of UGTs, the 44-amino acid conserved sequence of the PSPG box, was used as a query to search against peach genome database at the Phytozome (v11.0^2^) with BLASTP program to identify peach UGTs. Information of peach UGTs, including ORFs, description, and chromosome distribution were obtained from the same database. The distribution of *UGT* genes on the chromosomes was visualized with the MapChart (v2.3). The identification of signal peptide in UGT sequences was performed by SignalP 4.1 Server^3^.

### Sequence Alignment and Phylogenetic Analysis

The predicted amino acid sequences of *UGT* genes were aligned using the neighbor-joining (NJ) method in ClustalX v2.0 program. Phylogenetic tree was constructed with FigTree v1.4.2 program.

### Intron Mapping and Organization

The peach *UGT* intron map was constructed by determining the intron length, splice sites, phases, and positions in the genome. The exon–intron structure and intron phases were acquired by the online Gene Structure Display Server 2.0^4^. The intron can be inserted anywhere in the transcript, they are located either between codons or within codons which termed as intron phases, and the phases as well as location usually stay unchanged for a long time. Specifically, intron phases were determined as follows: introns positioned between two codons were defined as phase 0, introns positioned between first and second base of codon were defined as phase 1, and introns positioned between second and third base were defined as phase 2 (Barvkar et al., 2012).

### Gene Expression Analysis Using RNA-seq

RNA was extracted according to Zhang et al. (2006), and quality was monitored by gel electrophoresis and A260/A280. Libraries for high-throughput Illumina strand-specific RNA-seq were prepared as described previously (Zhang et al., 2016). Three biological replicates for various organs, fruit development and ripening stages and treatment were prepared. Transcript profiles for selected peach UGTs were obtained and expressed as heatmaps.

### Extraction of Glycosylated Bound Volatiles

Glycosylated bound volatiles were extracted according to Yauk et al. (2014) with modifications. Twenty grams of frozen samples were ground in liquid nitrogen, and were homogenized with water. After centrifuging for 20 min at 13,000 *g*, the supernatant was used as crude extract. Isolation of glycosidic precursors was conducted by using SPE LC-18 resins (CNW, Duesseldorf, Germany), which was previously preconditioned with methanol and distilled water. Eliminating free volatile compounds was performed by washing with dichloromethane, and the bound fraction was eluted with methanol. The bound volatile compounds were enzymatic hydrolyzed by adding AR2000 (Rapidase, Séclin, France) at 37°C for 48 h. The released volatiles were extracted with solid phase micro extraction, followed by identification using gas chromatography–mass spectrometry (GC–MS).

### GC–MS Analysis

Gas chromatograph (Agilent 7890N) coupled with Agilent 5973C mass spectrophotometer (Agilent, Palo Alto, CA, USA) were applied for identification of volatile compounds according to methods described by Zhang et al. (2010). Helium was used as a carrier gas at a flow rate of 1.0 mL min^-1^. The column effluent was ionized by electron ionization (EI) at energy of 70 eV with the transfer temperature of 250°C and the source temperature of 230°C. Mass scanning was done from 35 to 350 m/z with a scan time of 7 scans per second. Volatile compounds were identified by comparing with their EI mass spectra to NIST/EPA/NIH Mass Spectral Library (NIST-08 and Flavor) and retention time of authentic standards (Sigma-Aldrich, St. Louis, MO, USA) when available. Semi-quantitative determination of compounds was performed using the peak area of the internal standard as a reference based on total ion chromatogram (TIC) and calculated based on standard curves of authentic compounds.

### Protein Recombination of UGTs

Construction of expression plasmids of peach *UGT* genes were carried out by cloning the full-length ORFs with the N-terminal His-tag into the pET6xHN expression vector (Clontech, Palo, CA, USA). Primers for cloning were listed in Supplementary Table S1. The identity of the cloned gene was confirmed by sequencing of the complete insert. Recombinant proteins were heterologous expressed in *E. coli* BL21 (DE3) pLysS (Promega, Madison, WI, USA). The transformed cells were precultured at 37°C overnight in 20 mL Luria-Bertani (LB) medium containing 100 μg mL^-1^ ampicillin, and then inoculated into 1 L LB medium containing the same antibiotics. The culture was grown at 37°C at 150 rpm to an OD (600 nm) = 0.5 to 0.6, and the protein expression was induced with 1 mM isopropylthio-β-galactoside (IPTG). The culture was incubated at 16°C at 150 rpm for 20 h. The cells were harvested by centrifugation (4,000 *g*, 4°C, and 15 min) and resuspended in 1× PBS (1.37 M NaCl, 26.8 mM KCl, 20.3 mM Na_2_HPO4, and 17.6 mM KH_2_PO4, pH 7.2–7.4). After storing at -80°C overnight, the cells were disrupted by sonication. The supernatant was obtained by centrifugation (10,000 *g*, 4°C, and 30 min), and then purified using TALON Spin column (Clontech, Palo, CA, USA) following the manufacturer’s instructions.

### UGT Enzyme Activity Assay

Activity assays of recombinant peach UGTs were carried out in reactions using purified protein at final concentration at 0.4 μg μl^-1^. Reactions were performed in a buffer containing 100 mM Tris-HCl buffer (pH 7.5, and 2.0 mM DTT), 1.0 mM UDP-glucose (Sigma-Aldrich, St. Louis, MO, USA) and 1.0 mM substrate in a volume of 200 μl. Enzyme assays were incubated at 30°C for 16 h and terminated by addition of 1 μl 24% (*v/v*) TCA. The reaction mixture was extracted with ethyl acetate and then were evaporated to dryness. The glucosides were dissolved in methanol and analyzed by Agilent 1290 Infinity LC System (binary pump G4220A, diode array UV/VIS detector G4212A; Agilent Technologies, USA) coupled with a SunFire C18 analytical column (5 μm, 4.6 mm × 250 mm; Waters, USA). The column was operated at a temperature of 25°C. The chromatograms were obtained and detected between 200 and 400 nm. High performance liquid chromatography (HPLC) were performed at a flow rate of 1 mL min^-1^ with 100% water as solvent A and 100% acetonitrile as solvent B. The injection volume of samples was 20 μl. The column was firstly equilibrated with 10% solvent B, and eluted with a linear gradient program from 30 to 70% solvent B for 15 min, then washed with 100% solvent A for 15 min. The mass spectrometry analyses were performed by an Agilent6460 triple quadrupole mass spectrometer equipped with an ESI source (Agilent Technologies, USA) that operated in negative ionization mode. The scan range was 100–1000 m/z, and the nebulizer pressure was set as 45 psi. Identification of glucoside was based on the HPLC retention time and mass spectrometry spectral data. The glucoside of 2-phenylethanol was quantified by absorption at 210 nm, and the m/z value was 283 [M-H]^-^.

### Data Analysis

MultiExperiment Viewer (version 4.6.0) was applied for heatmap of peach *UGT* genes transcript abundance and for gene clusters construction. Correlation analysis between *UGT* genes transcript levels and contents of glycosylated bound volatile compounds was performed by MetaboAnalyst 3^5^.

## Results

### Identification and Phylogenetic Analysis of Peach UGTs

**FIGURE 1:**
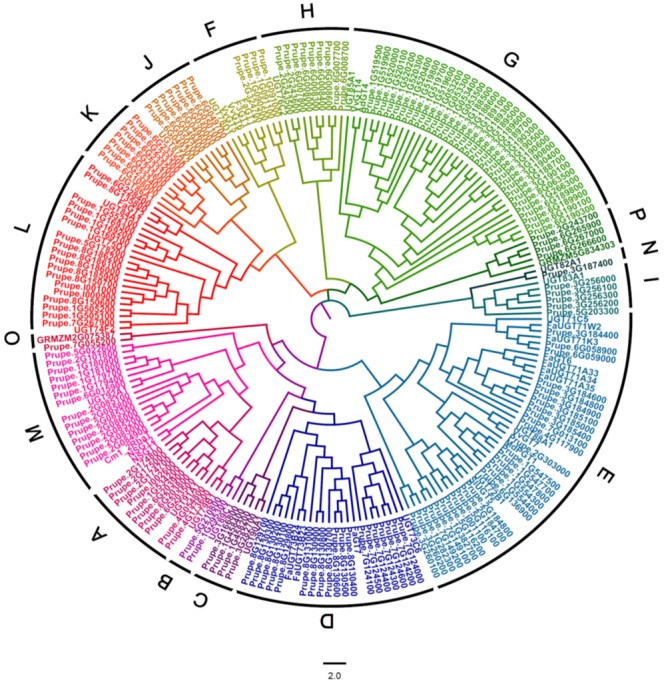
**Phylogenetic analysis of peach *UGT* family genes.** A phylogenetic tree was constructed by aligning the full-length amino acid sequences of peach UGTs with 19 Arabidopsis UGTs, 2 maize UGTs and several other UGTs that have been functionally characterized in fruit, including tomato, citrus, grapevine, apple, kiwifruit, and strawberry.

A BLASTP research of peach genome (Phytozome v11.0) was performed using UGT conserved PSPG box sequence as a query. A total of 168 peach UGTs having lengths of 150–616 amino acids were identified. A phylogenetic tree of peach *UGT* genes was constructed by aligning the full-length amino acid sequences of the peach UGTs with functionally characterized plant UGTs, including Arabidopsis, maize and UGTs from tomato, citrus, grapevine, apple, kiwifruit and strawberry (**Figure 1**). These peach UGTs were phylogenetically divided into 16 groups, including 14 groups (A–N) that were identified in Arabidopsis (Li et al., 2001). Two peach UGTs that were identified to be involved in metabolism of anthocyanin (Cheng et al., 2014) were clustered in group F, namely Prupe.1G091100 (PpUGT78A1) and Prupe.1G091000 (PpUGT78A2). Other UGTs responsible for glycosylation of anthocyanin were also located in group F, including strawberry *FaGT1* (Griesser et al., 2008), kiwifruit *F3GT1* (Montefiori et al., 2011), and grapevine *VvGT5* and *VvGT6* (Ono et al., 2010).

Distribution of plant UGTs in phylogenetic groups was summarized in **Table 1**. A total of 17 groups were detected in plants. Except for *Zea mays* (maize), no plant UGTs was detected in group Q. It is worth noting that group G had 34 UGTs from peach and 40 from apple, while 6–20 in other plants (**Table 1**). Moreover, group M contained 14 UGTs from peach and 15 in apple, compared with 1–5 in other species. These results indicated groups G and M may play important roles in the Rosaceae family such as apple and peach.

**Table 1:** Number of the plant UGTs in the different phylogenetic groups.

UGT group	*Arabidopsis thaliana*^a^	*Prunus persica*	*Malus x domestica*^a^	*Vitis vinifera*^a^	*Linum usitatissimum*^b^	*Oryza sativa*^a^	*Zea mays*^c^
A	14	10	33	23	16	14	8
B	3	2	4	3	5	9	3
C	3	4	7	4	6	8	5
D	13	19	13	8	21	26	18
E	22	29	55	46	22	38	34
F	3	4	6	5	1	–	2
G	6	34	40	15	19	20	12
H	19	9	14	7	6	7	9
I	1	5	11	14	9	9	9
J	2	7	12	4	4	3	3
K	2	7	6	2	5	1	1
L	17	18	16	31	19	23	23
M	1	14	13	5	3	5	3
N	1	1	1	1	1	2	4
O	–	1	5	2	–	6	5
P	–	4	5	11	–	9	1
Q	–	–	–	–	–	–	7
**Total**	**107**	**168**	**241**	**181**	**137**	**180**	**147**

*^a^Data from Caputi et al. (2012); ^b^Data from Barvkar et al. (2012); ^c^Data from Li et al. (2014).*

### Chromosome Distribution of Peach UGT Genes

**FIGURE 2:**
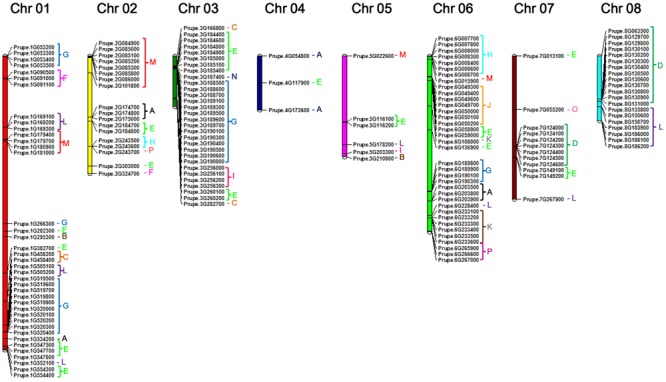
**Chromosome distribution of peach *UGT* genes.** The chromosome numbers were shown at the top of each chromosome. Letters of different colors indicate the different phylogenetic groups of peach *UGT* genes.

To provide an overview of location of peach *UGT* genes, genomic distribution of each UGT on the genome is shown in **Figure 2**. There are 164 *UGTs* distributed across all eight chromosomes of peach (**Figure 2**), while the left four UGTs located on scaffolds, including *Prupe.I000900, Prupe.I001000, Prupe.I001100*, and *Prupe.I002600* (Supplementary Table S2). Different members of UGTs were observed for each chromosome. There were 39 UGTs on chromosome 01, followed by 36 members on chromosome 06, and 30 on chromosome 03 (**Figure 2**). Only three UGTs were observed on chromosome 04.

Considering that peach *UGTs* could be separated into 16 groups, distribution of these groups on the chromosome were investigated (**Figure 2** and **Supplementary Figure S2**). Group G consisting of 34 peach UGTs had 15 members located on chromosome 1 and 14 on chromosome 03. For group D, 19 members were identified in peach (**Table 1**), with 7 and 12 UGTs located on chromosomes 07 and 08, respectively. On the contrary, *UGTs* of group E are randomly distributed across seven chromosomes (01–07) (**Figure 2** and **Supplementary Figure S2**).

### Intron and Exon Structure of Peach UGT Genes

To investigate the evolutionary relationships within peach *UGT* gene family, the exon–intron organization was analyzed. Among the 168 peach *UGTs*, 72 had no introns, 82 contained one intron each in them (**Table 2**). For the remaining 14 *UGTs*, nine had two introns, four had three introns, and one had five introns. For *UGT* groups, the largest numbers of genes losing introns was observed for group E with 23 members, followed by 16 in group D and 13 in group M. A total of 31 (91%) *UGTs* in group G contained one intron, followed by 12 in group L (**Table 2**).

**Table 2:** Number of peach *UGT* genes in each group according to introns amount.

No. of introns Group	0	1	2	3	5	Total
A	7	1	1	0	1	**10**
B	2	0	0	0	0	**2**
C	2	0	2	0	0	**4**
D	16	1	1	1	0	**19**
E	23	4	1	1	0	**29**
F	1	2	1	0	0	**4**
G	1	31	1	1	0	**34**
H	0	8	0	1	0	**9**
I	0	5	0	0	0	**5**
J	0	5	2	0	0	**7**
K	0	7	0	0	0	**7**
L	6	12	0	0	0	**18**
M	13	1	0	0	0	**14**
N	0	1	0	0	0	**1**
O	1	0	0	0	0	**1**
P	0	4	0	0	0	**4**
**Total**	**72**	**82**	**9**	**4**	**1**	**168**

**FIGURE 3:**
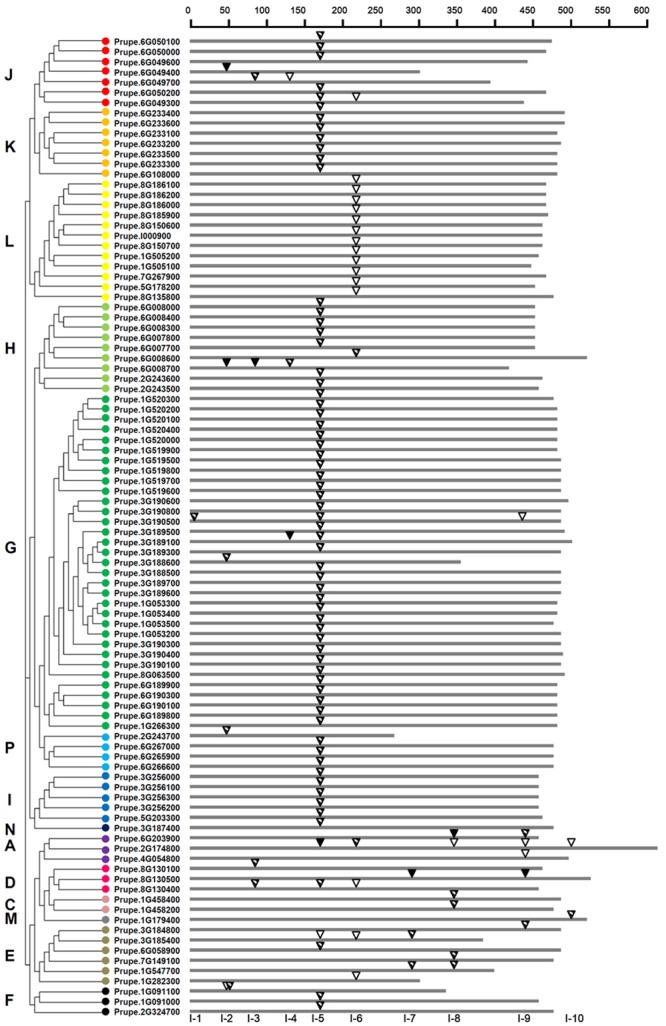
**Distribution of introns among 96 *UGT* genes in peach.** The introns were mapped and numbered to the alignment of the amino acid sequences encoded by the *UGT* genes. The scale on the top of map shows the intron insertion on each gene. The gray thick lines indicate the length of the amino acid residues. Inverted triangles indicate the positions of introns occurred on each gene. Intron phases are indicated by open inverted triangles (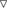), slash filled inverted triangles (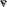), and black inverted triangles (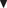) for 0, 1, and 2, respectively. The classification of peach *UGT* genes was indicated by the phylogenetic relationship on the left, and different phylogenetic groups were distinguished by the colored dots.

A total of 96 peach *UGTs* containing intron sequences was observed. After mapping the introns to the amino acid sequences alignment, at least 10 independent intron insertion events were observed. These insertion events are serially numbered as I-1 to I-10 according to their positions (**Figure 3**). The high conserved introns were observed for I-5 (intron 5), containing 66 (69%) peach *UGTs* belonging to group A, D–K, N, and P. Among these groups, all members of group K, I, and N contained the intron 5. For group G, 32 out of 33 UGTs had high conserved intron 5. The intron 6, was predominantly observed in group L.

Among the total 118 introns detected in peach *UGTs* sequences, 25, 84, and 9 were in phase 0, 1, and 2, respectively (**Figure 3** and Supplementary Table S3). For the high conserved intron 5, only one was in phase 0, one was in phase 2, and phase 1 accounted for 97% of all introns (**Figure 3**). For intron 6, 16 out of 18 *UGTs* were in phase 0. This suggests that the high conserved introns were in the same intron phase.

### Tissue Specificity and Temporal Expression Patterns in Fruit during Development and Ripening

**FIGURE 4:**
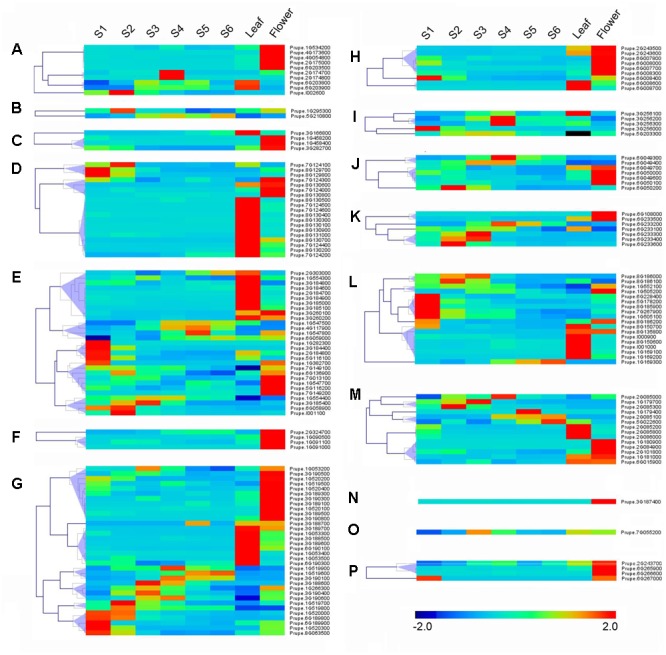
**Expression profiles of peach *UGT* genes in different organs, and during peach development and ripening.** The peach *UGT* genes, classified into different groups, expressed in different organs, including flowers, leaves, and fruits. The heat maps were analyzed by hierarchical clustering of *UGT* genes. The color scale (representing –2 to 2) is shown. **(A–P)** means different phylogenetic groups of peach *UGT* genes.

RNA-seq was carried out to examine the expression pattern of 168 peach *UGTs* in flowers, leaves, and during fruit development and ripening. A total of 45 (27%) *UGTs* showed the highest levels of transcripts in leaf (**Figure 4**). For group D, 12 out of 19 *UGTs* were predominantly expressed in leaf. For peach flower, totally 54 (32%) *UGTs* had the highest expression. All members of groups F and N, and more than half members of group A, C, H, J, and P accumulated the most abundance of UGTs transcripts in flower (**Figure 4**). Tissue-specific expression of *Prupe.1G091100* and *Prupe.1G091000* in group F were consistent with previously study, where these genes were involved in biosynthesis of peach flower anthocyanin (Cheng et al., 2014). A total of 60 *UGTs* had the highest abundance in developing fruit and post-harvest ripening. These genes primarily expressed in fruit were from 13 groups of peach UGT except for group C, F, and N (**Figure 4**).

Among fruit-specific expression of these peach *UGT* genes, transcript levels of 19 (35%) members in peach was the highest at S1 (the first fast growth), followed by 11 at S2 (stone hardening), nine at S3 (the second fast growth), and 10 at S4 (mature stage). After fruit harvested at S4, expression of peach *UGTs* usually tended to decrease during shelf-life at ambient temperature (**Figure 4**). Considering that group G had the largest number of *UGT* genes in peach, expression patterns during fruit ripening was further analyzed. Five members of UGTs showed the highest transcript levels at S1 stage, including *Prupe.1G520000, Prupe.6G189800, Prupe.6G189900, Prupe.1G520300*, and *Prupe.8G063500*. Transcript levels of three *UGTs* peaked at S4 stage during fruit development, while one member further accumulated during post-harvest ripening at S6 stage (**Figure 4**).

### Expression of Peach UGTs in Response to UV-B Irradiation

**FIGURE 5:**
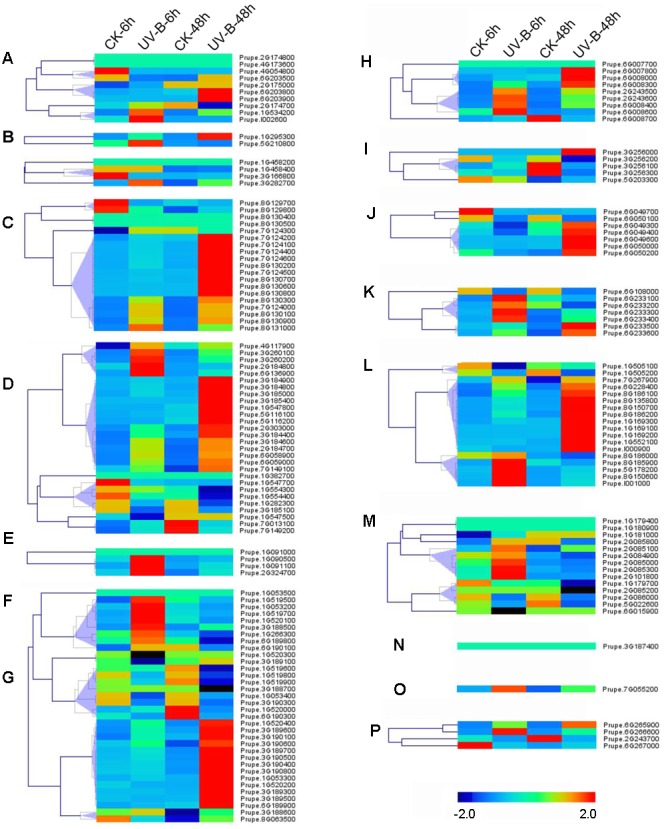
**Expression profiles of peach *UGT* genes in response to UV-B treatment.** Peach fruits at S4 stage were treated with UV-B for 6 and 48 h, and each treatment has a corresponding control, show as 6 and 48h-CK, respectively. The heat maps were analyzed by hierarchical clustering of *UGT* genes. The color scale (representing –2 to 2) is shown. **(A–P)** means different phylogenetic groups of peach *UGT* genes.

Abiotic stresses such as UV-B irradiation can affect production of secondary metabolites (Kaling et al., 2015), some of which such as phenylpropanoid are primarily presented as glycosylated form due to UGTs activity (Roy et al., 2016). To test if UV-B treatment could alter expression of *UGT* genes, transcript profiles were investigated in peach fruit (**Figure 5**). After 6 h irradiation, transcripts of group F *Prupe.1G091100* was induced. Similar pattern was also observed for *Prupe.1G090500* and *Prupe.2G324700* in group F, five members in groups E and M each, four members in groups H, K, and L each. Transcript levels of 65 (39%) *UGT* genes were significantly induced after 48 h UV-B treatment, including nine members in Group D, 14 in Group E, 13 in Group G, 5 in Group J, and 10 in Group L (**Figure 5**).

### Identification of Peach UGTs Associated with Glycosylated Bound Volatile Formation

**FIGURE 6:**
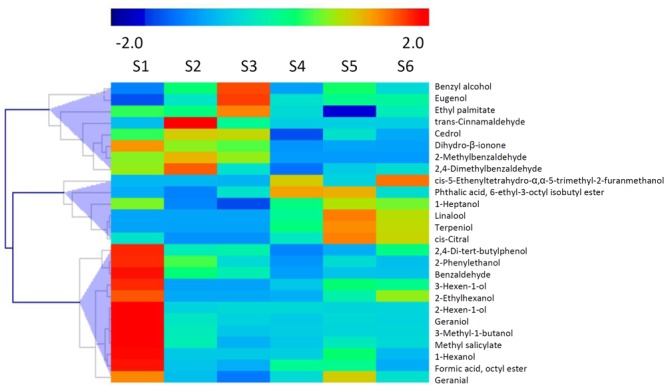
**Changes in content of glycosylated volatiles during peach fruit development and ripening.** The heat map was analyzed by hierarchical clustering of glycosylated volatiles. The color scale (representing –2 to 2) is shown.

It has been reported that glycosylated bound volatile was a potential source for aroma formation, and formation of these non-volatile compounds were catalyzed by UGTs via transferring activated sugar molecules to volatile compounds (Schwab et al., 2015). A total of 26 glycosylated volatiles were identified in peach fruit during development and ripening (**Figure 6**). These chemicals composed of glycosylated aldehydes, esters, ketones and alcohols, including methyl salicylate, C6 alcohols, linalool and 2-phenylethanol. The detected glycosylated volatiles are clustered into three groups based on changes in content during fruit development and ripening. The first group consisted of 12 compounds, including 2-phenylethanol, which had the highest content at S1 stage (**Figure 6**). For the second group of glycosylated volatiles, content of benzyl alcohol and eugenol increased and peaked at S2 or S3 stage. Glycosylated linalool and terpeniol belong to the third group, which had the highest content at S5 stage. To mine *UGT* genes that are associated with glycosylated bound volatile formation, a correlation analysis between levels of transcripts and contents of volatiles was carried out (**Supplementary Figure S3**). A total of 128 (76%) members had positive correlation with the glycosylated volatiles detected in peach fruits.

**FIGURE 7:**
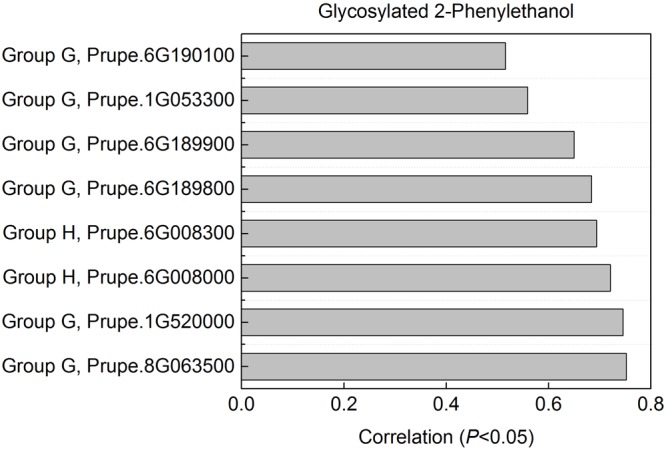
**Correlation between *UGT* genes with glycosylated 2-phenylethanol.** For glycosylated 2-phenylethanol, eight peach *UGTs* had significant positive correlation were shown. Expression profile of the eight UGT genes during peach fruit development and ripening were shown in **Figure 4**.

**FIGURE 8:**
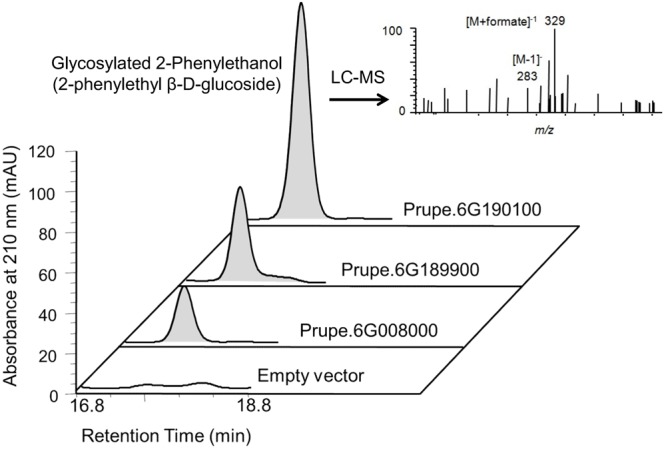
**High performance liquid chromatography (HPLC) and LC–MS analysis of glycosylated 2-phenylethanol catalyzed by peach UGTs.** Using UDP-glucose as sugar honor and 2-phenylethanol as substrate, products catalyzed by peach three UGTs were analyzed by HPLC. The empty vector (pET6xHN) was shown on the bottom as control. LC–MS analysis showed the mass spectra of 2-phenylethanol β-D-glucoside consisted of a parent molecule ion [M-H]^-^ with mass/charge (m/z) 283 [283 = 122 (2-phenylethanol) + 162 (Glc – water) – H]^-^, and its formic acid adduct m/z 329 [329 = 283 + 46(formic acid)].

2-phenylethanol was an important volatile compound derived from phenylalanine that contribute to fruit overall flavor quality (Tieman et al., 2006), and then was used to further analysis. There are eight peach *UGTs* showing significant positive correlation with 2-phenylethanol (**Figure 7**), therefore, their enzyme activity was analyzed. Recombinant proteins were obtained via heterologous overexpressing these eight *UGTs* in *E. coli*. As shown in **Figure 8**, Prupe.6G008000, Prupe.6G190100, and Prupe.6G189900 could transfer the UDP-glucose to 2-phenylethanol. Regarding the other five UGTs, no activity was observed for recombinant proteins toward 2-phenylethanol. These results suggested that these three UGTs Prupe.6G008000, Prupe.6G190100, and Prupe.6G189900 were likely to be associated with formation of 2-phenylethyl β-D-glucoside in peach fruit. Subcelluar localization of these three active UGTs was predicated using SignalP program, and no signal peptides were observed. These observation is consistent with the general assumption that plant UGTs are cytoplasmic enzymes (Li et al., 2001).

## Discussion

Plant UGTs play an important role in regulating of metabolic homeostasis, detoxification of xenobiotics, and the biosynthesis, storage and transport properties of secondary metabolites (Yonekura-Sakakibara and Hanada, 2011). The *UGT* multigene family has been identified in several plant species, including Arabidopsis (Caputi et al., 2012) and rice (Moon et al., 2013), fruit species such as grape (Bönisch et al., 2014a,b), kiwifruit (Yauk et al., 2014), and strawberry (Song et al., 2016a,b). Peach was considered as a model plant of Rosaceae family (Shulaev et al., 2008), however, only two UGTs were characterized to be associated with anthocyanin metabolism (Cheng et al., 2014). Thus, it is essential to extend UGTs study to peach fruit.

In the present study, 168 *UGT* genes were identified in peach and were clustered into 16 groups based on phylogenetic analysis. *UGT* genes in peach account for approximately 0.6% of the gene products in peach (Verde et al., 2013), higher than 0.4% in chickpea (Sharma et al., 2014). Highly conserved 14 groups (A–N) in plants and two newly discovered groups O and P were observed in peach (**Table 1**). Combination with previous reports (Li et al., 2014), there are at 10 plant species containing groups O and P, including peach in the present study. It is worth noting that expansion of group N was only observed in monocots such as maize and rice, while only one member was detected in dicot plants (**Table 1**). In addition, the larger number of *UGT* genes in peach than Arabidopsis (107 members) is due primarily to an expansion within the groups G and M. Groups G and M contained 34 and 14 members, accounting for 20 and 8% of *UGT* genes in peach, respectively. Interestingly, among the plant species analyzed, groups G and M only expanded in peach and apple, and there was eight and one member in Arabidopsis, respectively (Caputi et al., 2012). These results implied these two groups may play important roles in glycosylation of small molecules in the Rosaceae family although more detailed research is required.

From a general point of view, intron gain and loss events, as well as the positions and phases of introns relative to protein sequence are important cues in understanding evolution (Rogozin et al., 2000). Intron mapping of 168 peach *UGTs* revealed that 43% members lacked introns, which is less than the number (60%) of maize (Li et al., 2014) and the number (58%) of Arabidopsis *UGT* genes (Li et al., 2001), while close to the number (40%) for flax (Barvkar et al., 2012). Ten intron positions were identified in peach *UGT* genes, with I-5 (intron 5) being the most widespread intron (**Figure 3**). Intron 5 was observed in most members of groups G–L and P, and was regarded as the oldest intron in peach *UGTs*. In maize 147 *UGTs*, intron 5 was also considered as the oldest intron (Li et al., 2014). The second highly conserved intron was observed for intron 6 that was predominantly observed in group L of peach. It is worth noting that the plenty of intron 5 were in phase 1, suggesting that majority of conserved introns were ancient elements and their phases remain stable (Roy and Gilbert, 2005).

To investigate the function of peach *UGT* genes, expression analysis by RNA-Seq were conducted in various tissues, during fruit development and ripening, and in response to abiotic stresses such as UV-B irradiation. In agreement with previous study (Cheng et al., 2014), *Prupe.1G091100* and *Prupe.1G091000* in group F were mainly expressed in peach flowers. These two UGTs have been reported to be responsible for synthesis of anthocyanin of peach flower (Cheng et al., 2014). For fruit-specific *UGT* genes, totally 35% *UGT* genes had the highest transcript levels in S1 stage. Peach fruit development was divided into four distinct stages, in which S1 (approximately 23–37 DAB) was the first exponential growth phase and was characterized by a rapid cell division (Lombardo et al., 2011). High abundance of peach UGTs transcripts in intense cell division of S1 stage was also observed in other plants such as Arabidopsis root apex, lateral root initials and leaf periphery (Woo et al., 2007), and chickpea germinating seeds and flower (Sharma et al., 2014). These results suggested that localization of plant UGTs in areas of intense cell division was possibly involved in cell cycle regulation.

Furthermore, post-harvest UV-B irradiation induced transcript accumulation of most peach *UGTs*, including groups D, E, F, G, H, J, and L. Given that UV-B is involved in regulation of metabolic rearrangements in plants (Kaling et al., 2015) and UGTs are associated with secondary metabolites metabolism, we proposed that changes in *UGTs* expression may be responsible for glycosylation of metabolites in response to UV radiation. It has been long known that anthocyanins are usually present as glycosylated forms, and serve to protect plant against stresses such as UV radiation (Winkel-Shirley, 2001). Accelerated accumulation of anthocyanins in response to UV-B has been observed in fruit such as nectarine (Ravaglia et al., 2013) and peach (Scattino et al., 2014). In the present study, induced transcript levels of *Prupe.1G091100* in group F by UV-B treatment coincided with observation that this *UGT* gene was responsible for formation of glycosylated anthocyanins in peach (Cheng et al., 2014). Therefore, these results suggested that peach *UGTs* may play an important role in response to UV irradiation.

As mentioned above for anthocyanins, glycosylation is also a major regulator of phenylpropanoids availability and biological activity in plants (Roy et al., 2016). Consistent with previous studies in fruit such as grape (Bönisch et al., 2014a,b) and kiwifruit (Yauk et al., 2014), glycosylated aldehydes, alcohols and esters derived from phenylalanine pathway were also detected in ripening peach fruit. Among these compounds, 2-phenylethanol has pleasant fruity odor in ripening fruit, and has been known due to their attractiveness to mammals and other seed dispersers (Tieman et al., 2006). The expression profiles of eight *UGT* genes significantly correlated with the decreased patterns of the glycosylated 2-phenylethanol (**Figure 7**). Three of the eight recombinant UGT proteins tested, Prupe.6G008000, Prupe.6G190100 and Prupe.6G189900, were observed to be involved in the *in vitro* biosynthesis of glycosylated 2-phenylethanol. The phylogenetic analysis showed that *Prupe.6G189900* and *Prupe.6G190100* belong to group G, and were homologous to kiwifruit *AdGT4* and grape *VvGT14*. It has been reported that *AdGT4* and *VvGT14* were responsible for glycosylation of 2-phenylethanol (Bönisch et al., 2014a; Yauk et al., 2014). These results indicated that peach *UGT* genes were involved in the biosynthesis of flavor-related volatiles during fruit development and ripening.

## Conclusion

Peach contains 168 *UGT* genes distributed across eight chromosomes. These genes were clustered into 16 groups based on phylogenetic analysis, in which groups O and P were newly detected in plants. Totally 10 intron positions were identified in peach *UGT* genes, indicating their divergence and evolutionary relationships between UGTs. RNA-seq analysis revealed tissue-specific expression in leaf, flower and fruit. Furthermore, changes in transcript levels were detected during fruit development and ripening, and in response to abiotic stress, suggesting essential roles of *UGTs* in peach. Glycosylated volatile compounds were detected in developing fruit, whose contents correlated with transcript levels of peach *UGTs*. Heterologous expressing in *E. coli* revealed three *UGT* genes were involved in the *in vitro* biosynthesis of glycosylated 2-phenylethanol.

## Author Contributions

BW and BZ designed the whole experiments, analyzed the data, and wrote the paper; LG, JG, YX, HL, and XC performed the experiments; KC contributed reagents, materials, and analysis tools.

## Conflict of Interest Statement

The authors declare that the research was conducted in the absence of any commercial or financial relationships that could be construed as a potential conflict of interest.

## Footnotes

1 http://www.cazy.org

2 https://phytozome.jgi.doe.gov/

3 http://www.cbs.dtu.dk/services/SignalP-4.1/

4 http://gsds.cbi.pku.edu.cn/

5 http://www.metaboanalyst.ca/

## Supplementary Material

FIGURE S1 **Peach fruit growth curve and photos of four stages**.

FIGURE S2 **The number of *UGT* genes belonging to different phylogenetic groups on each chromosome**.

FIGURE S3 **Correlation matrix between *UGT* genes with glycosylated bound volatile compounds**.
