# Supplementary figures and images for: Genome-Wide Identification, Expression Patterns, and Functional Analysis of UDP Glycosyltransferase Family in Peach (Prunus persica L. Batsch)
Source: Front Plant Sci. 2017 Mar 22;8:389. doi: 10.3389/fpls.2017.00389 (PMC5360731; doi:10.3389/fpls.2017.00389)

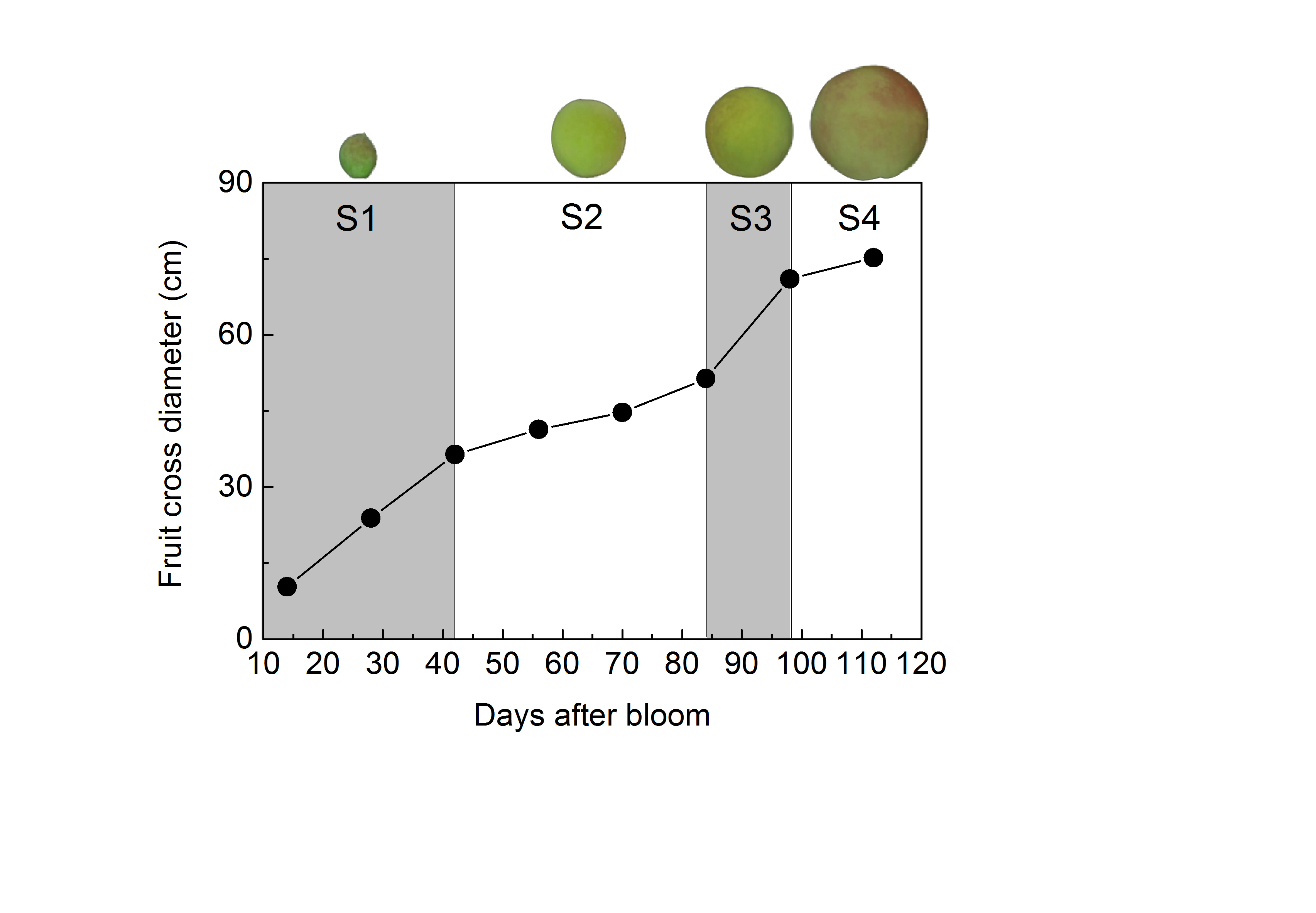

Supplement: FIGURE S1 — Peach fruit growth curve and photos of four stages. [file Image_1.JPEG]

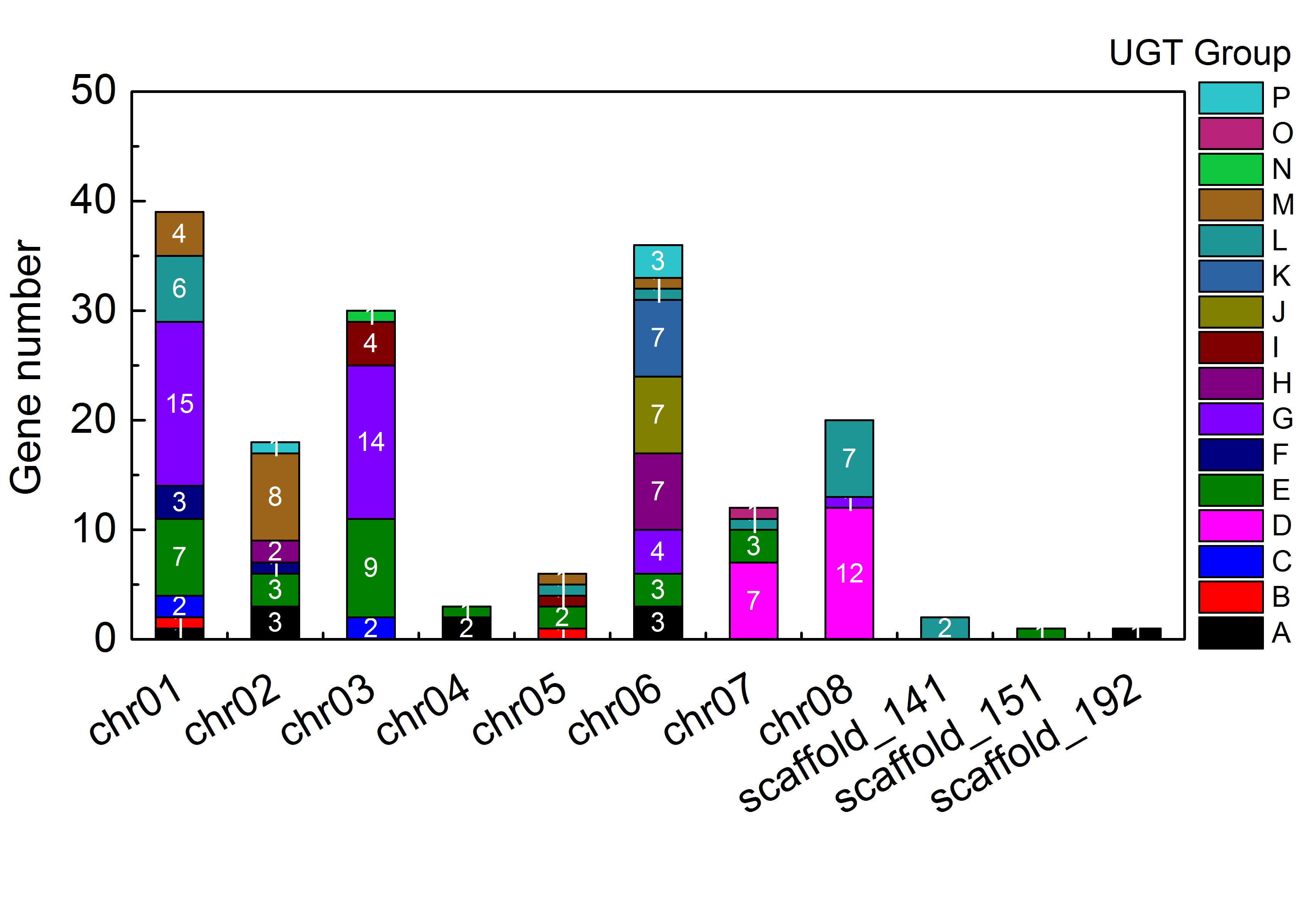

Supplement: FIGURE S2 — The number of UGT genes belonging to different phylogenetic groups on each chromosome. [file Image_2.JPEG]

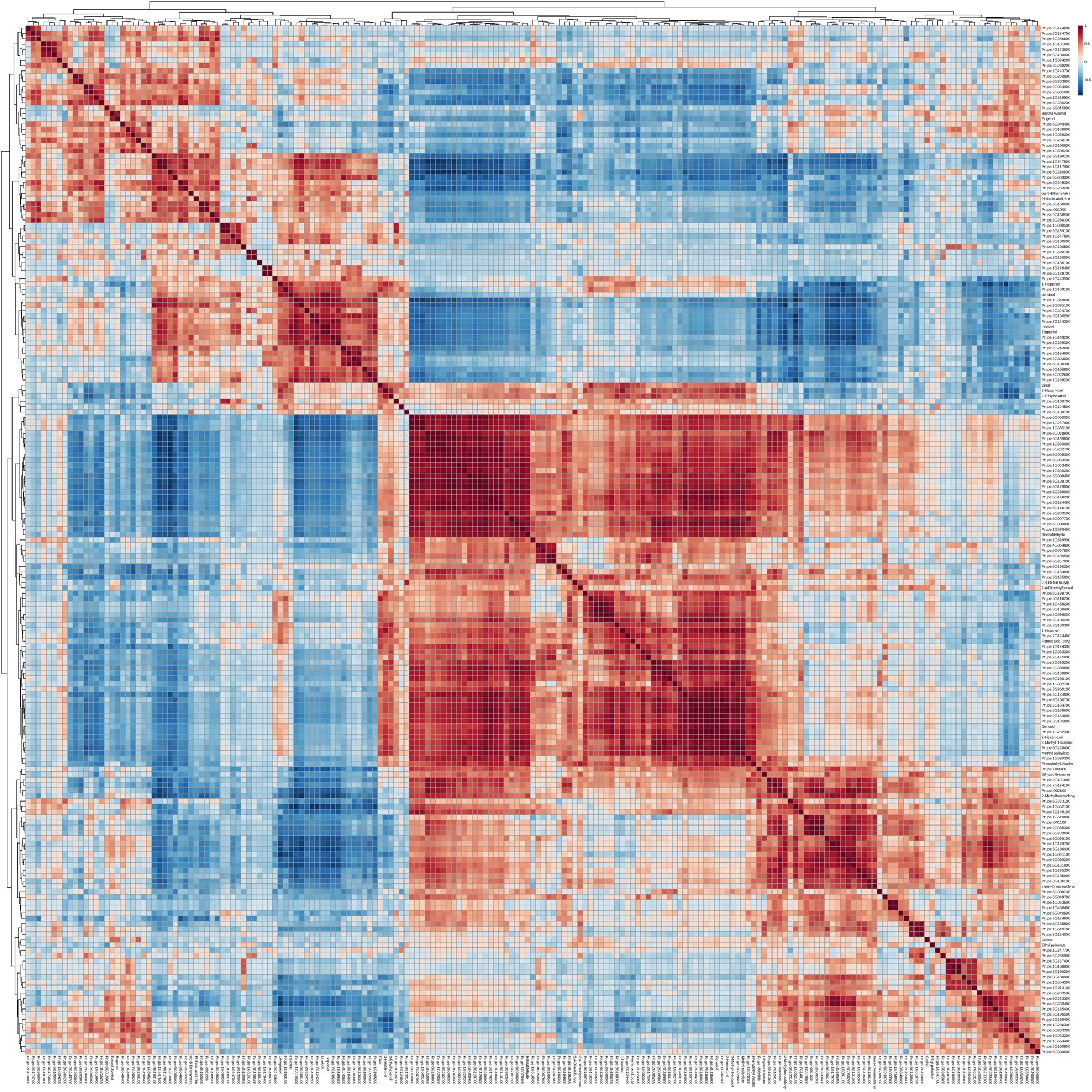

Supplement: FIGURE S3 — Correlation matrix between UGT genes with glycosylated bound volatile compounds. [file Image_3.JPEG]
